# Managing Hypertension in Chronic Kidney Disease: The Role of Diet and Guideline Recommendations

**DOI:** 10.3390/jcm14113755

**Published:** 2025-05-27

**Authors:** Emelina Stambolliu, Panagiotis Iliakis, Konstantinos Tsioufis, Aikaterini Damianaki

**Affiliations:** 1 Nephrology Department, Hippokration General Hospital, 11527 Athens, Greece; 2 First Department of Cardiology, School of Medicine, National and Kapodistrian University of Athens, Hippokration General Hospital, 11527 Athens, Greece; panayiotisiliakis@gmail.com (P.I.);

**Keywords:** hypertension, chronic kidney disease, dietary interventions, sodium, potassium

## Abstract

Lifestyle and dietary modifications are unanimously suggested as the initial step to treat hypertension in the general population and in patients with chronic kidney disease (CKD). Limiting sodium intake constitutes the cornerstone of dietary interventions, but augmenting dietary potassium intake has also been associated with a significant blood pressure (BP)-lowering effect. Although there may be a consensus about restraining the daily sodium intake to <2 g per day, the target for optimal potassium intake is vague. In hypertensive patients with CKD, the desired amount of potassium in the diet remains a controversial issue, as evidence from studies concerning the effect on CKD progression is contradictory. Hence, medical societies and food authorities worldwide do not share a joint recommendation. Other dietary components, including calcium, magnesium, protein, phosphorus, zinc, and alcohol intake may play a role in BP control, but the evidence in the CKD population so far is inconclusive. Further studies are needed to establish solid evidence about the safety and efficacy of dietary interventions, particularly in CKD patients, the majority of whom suffer from hypertension. The purpose of this review is to summarize the existing recommendations and evidence concerning dietary interventions in hypertensives with CKD, with a primary focus on sodium and potassium intake. Additionally, we briefly address other dietary components that may play a role in BP regulation or kidney function.

## 1. Introduction

Hypertension is a major global health challenge and is also highly prevalent in individuals with chronic kidney disease (CKD) [1]. These two conditions often reinforce each other and create a vicious cycle that accelerates kidney damage and increases the cardiovascular (CV) risk. Effective management of hypertension is crucial in slowing CKD progression and improving long-term outcomes [2]. While pharmacologic treatments are essential to control blood pressure (BP), lifestyle changes are also considered an indispensable tool for BP control even among this special group of patients. Several international guidelines highlighted a list of lifestyle interventions recommended in all hypertensive patients, including those with CKD [3,4]. Hence, implementing a low-sodium diet (with the exception of patients with salt-wasting nephropathy), managing weight excess, reducing alcohol intake to close to abstinence, increasing potassium consumption (except for patients with advanced CKD), and following dietary patterns such as the Dietary Approaches to Stop Hypertension (DASH) diet are considered as the initial steps of the management of hypertensive CKD patients too.

The Achilles’ heel of treatment strategies based on non-pharmacological interventions is the low persistence of the prescribed measures. Hence, adherence to dietary changes remains a challenge, especially in CKD patients, who face multiple barriers, including phycological distress, altered taste, limited access to fresh foods, and comorbidities [5,6,7,8].

This manuscript investigates the existing evidence concerning the effectiveness of various dietary interventions in hypertensive CKD patients.

## 2. International Medical Societies Recommendations on Salt and Potassium Intake

Medical societies around the world provide structured, evidence-based recommendations on the implementation of lifestyle interventions, including dietary modification, weight reduction, and physical activity enhancement, for preventing or delaying hypertension, which are even more important in patients with CKD. The most important recommendations (for patients with and without CKD) are presented in Table 1.

The recent 2023 European Society of Hypertension (ESH) Guidelines for the management of arterial hypertension highlight the importance of lifestyle modification towards reducing hypertension burden [3]. A healthy diet, such as the Mediterranean or the DASH diet, is at the center of the recommendations, with the DASH diet providing the strongest evidence of BP-lowering benefit [9,10,11]. Regarding salt intake, adults with hypertension who consume a high-sodium diet are recommended to replace part of sodium chloride (NaCl) with potassium chloride (KCl). Additionally, a universal salt restriction to less than 5 g/day (optimal 2 g/day) is advised for all hypertensive individuals; this restriction has been associated with a mean 5.4/2.8 mmHg (systolic/diastolic BP) reduction in hypertensives [12]. As far as potassium intake is concerned, an increased dietary potassium intake is recommended, with caution in patients with advanced CKD.

Recently, the European Society of Cardiology (ESC) published the 2024 ESC Guidelines for the management of elevated BP and hypertension, with equivalent suggestions [13]. They do not differ from the 2023 ESH Guidelines regarding dietary and salt recommendations, setting the same targets for daily salt intake. On the other hand, regarding potassium intake, they suggest increasing daily potassium intake by 0.5–1.0 g/day for hypertensive individuals without moderate to advanced CKD; for patients with CKD or under medical treatment associated with decreased potassium excretion (e.g., mineralocorticoid receptor antagonists), close monitoring of serum potassium levels is suggested. In addition, the American College of Cardiology (ACC)/American Heart Association (AHA) 2017 Guidelines for the prevention, detection, evaluation, and management of high BP in adults shares similar suggestions regarding non-pharmacological treatment via beneficial dietary implementation, as well as optimizing potassium and sodium intake [14]. Moreover, they emphasize an expected systolic BP reduction associated with weight loss (1 mmHg for every 1 kg reduction), DASH diet (optimal adherence to the diet may lead to 11 mmHg reduction of systolic BP), sodium intake (targeting a diet with <1.5 g/day may lead to 5 mmHg reduction of systolic BP), and potassium intake (daily potassium intake of 3500–5000 mg, may lead to 4 mmHg reduction of systolic BP) [15,16,17,18]. In these guidelines, there are no CKD-specific dietary recommendations. Regarding recommendations from the Korean Society of Hypertension, both the 2018 and the 2022 focused update of the 2018 Korean Hypertension Society Guidelines for the management of hypertension align with the European and American medical societies in their recommendations for lifestyle and dietary modification in hypertensives with CKD [19,20]. In fact, they specifically recommend maintaining a healthy weight (BMI 20 to 25 kg/m^2^), reduction of sodium intake to <90 mmol (<2 g) per day unless contraindicated, engaging regular exercise with proven CV benefit, ceasing smoking, and limiting alcohol consumption. Specific mention could be made for older individuals and frail patients with hypertension and chronically deteriorated renal function, whose risk of orthostatic hypotension is increased, and the potassium restriction advice could be more lenient [21].

The 2021Kidney Disease Improving Global Outcomes (KDIGO) Clinical Practice Guideline on the Management of Blood Pressure in Chronic Kidney Disease provides specific recommendations on lifestyle modifications to reduce elevated BP in patients with CKD [4]. Specifically, a maximum of 2 g of sodium or 5 g of NaCl per day is recommended in patients with CKD and hypertension, while, in patients with CKD and sodium-wasting nephropathy, the restriction of dietary sodium intake is not evidently advised. Regarding recommended diets in patients with CKD, the guidelines state that the DASH diet or the use of potassium-enriched salt substitutes could be harmful for patients with advanced CKD, hyporeninemic hypoaldosteronism, or other causes causing impaired potassium excretion. Recently, KDIGO published the 2024 KDIGO Clinical Practice Guideline for the Evaluation and Management of Chronic Kidney Disease [22]. As anticipated, there are no major differences in the recommended daily sodium intake in CKD patients. However, there are some additional recommendations regarding potassium levels, the role of both pharmacological and non-pharmacological interventions, and the support of patients through advice and education from a renal dietitian. Dietitians should provide clear counselling on adapting the daily intake of foods rich in bioavailable potassium, especially for patients with CKD G3–G5 with a history of hyperkalemia. The aforementioned points are also highlighted in the National Kidney Foundation’s Kidney Disease Outcomes Quality Initiative (KDOQI) Commentary on the KDIGO 2024 Clinical Practice Guideline for the Evaluation and Management of CKD [23]. Regarding both dietary salt and potassium recommendations, KDOQI Clinical Practice Guideline for Nutrition in CKD: 2020 Update and KDOQI Commentary in the KDIGO 2024 align with the KDIGO 2024 Guidelines [23,24]. Furthermore, it is important to consider region-specific guidelines, such as those from the Asian Pacific Society of Nephrology (APSN). While largely consistent with international recommendations, APSN suggests a lower target BMI of 18–23 kg/m^2^ for individuals with CKD, in contrast to KDIGO and other societies [25]. However, sodium intake thresholds remain universally set at <2 g of sodium or <5 g of NaCl per day. Recently, the American Diabetes Association (ADA) has released the annual Standards of Care in Diabetes for 2025, which underscores sodium restriction (to <2 g/day) and individualized potassium intake in CKD patients to aid in BP management and CV risk reduction—factors that are particularly critical in individuals with impaired renal function [26].

As this manuscript focuses on dietary interventions and lifestyle modifications in hypertensive individuals with CKD, pharmacological treatment recommendations are beyond its scope.

**Table 1:** Summary of recommendations from medical societies on dietary interventions, including salt and potassium intake, in hypertensive patients [3,4,13,14,25].

Society	Recommendations	CoR	LoE
ESH (2023)	Preferred dietary products include vegetables, fruits, beans, nuts, seeds, vegetable oils, and fish and poultry among meat products. Fatty meats, full-fat dairy, sugar, sweetened beverages, and sweets should be limited. Overall, a healthy dietary pattern including more plant-based and less animal-based food is recommended.	I	B
	In adults with hypertension consuming a high-sodium diet (most Europeans), salt substitutes replacing part of the NaCl with KCl is recommended to reduce BP and the risk for CVD.	I	A
	Dietary salt (NaCl) restriction is recommended for adults with elevated BP to reduce BP. Salt (NaCl) restriction to <5 g (~2 g sodium) per day is recommended.	I	B
	Increased potassium consumption, preferably via dietary modification, is recommended for adults with elevated BP, except for patients with advanced CKD.	I	B
ESC (2024)	Adopting a healthy and balanced diet, such as the Mediterranean or DASH diets, is recommended to help reduce BP and CVD risk.	I	B
	Restriction of sodium to approximately 2 g per day is recommended where possible in all adults with elevated BP and hypertension (this is equivalent to about 5 g of salt ([14] per day or about a teaspoon or less).	I	A
	In patients with hypertension without moderate to advanced CKD and with high daily sodium intake, an increase of potassium intake by 0.5–1.0 g/day—for example through sodium substitution with potassium-enriched salt (comprising 75% NaCl and 25% KCl) or through diets rich in fruits and vegetables—should be considered.	IIa	A
	In patients with CKD or taking potassium-sparing medication, such as some diuretics, ACE inhibitors, ARBs, or spironolactone, monitoring serum levels of potassium should be considered if dietary potassium is being increased.	IIa	C
ACC/AHA/ AAPA/ ABC/ACPM/ AGS/APhA/ ASH/ASPC/ NMA/PCNA (2017)	A heart-healthy diet, such as the DASH diet, that facilitates achieving a desirable weight is recommended for adults with elevated BP or hypertension.	I	A
	Sodium reduction is recommended for adults with elevated BP or hypertension.	I	A
	Potassium supplementation, preferably in dietary modification, is recommended for adults with elevated BP or hypertension, unless contraindicated by the presence of CKD or use of drugs that reduce potassium excretion.	I	A
KDIGO (2021)	Targeting a sodium intake <2 g of sodium per day (or <90 mmol of sodium per day, or <5 g NaCl per day) in patients with high BP and CKD.	II	C
KDIGO (2024)	Targeting a sodium intake <2 g of sodium per day (or <90 mmol of sodium per day, or <5 g NaCl per day) in patients with high BP and CKD.	II	C
	A protein intake of 0.8 g/Kg body weight/day in adults with CKD G3–G5 is suggested.	II	C
APSN (2020)	Sodium restriction (<2 g of sodium per day or <90 mmol of sodium per day, or <5 of NaCl per day) in individuals with high BP and CKD with or without diabetes.	II	C

ESH, European Society of Hypertension; ESC, European Cardiology Society; KDIGO, Kidney Disease Improving Global Outcome; BP, blood pressure; CVD, cardiovascular disease; CKD, chronic kidney disease; DASH, Dietary Approaches to Stop Hypertension; APSN, Asian Pacific Society of Nephrology; ACC/AHA, American College of Cardiology (ACC)/American Heart Association (AHA); CoR, class of recommendation; LoE, level of evidence.

## 3. Sodium Reduction

Several studies in the general population have examined the effect of sodium restriction on BP and the risk of CV disease. Indeed, a high sodium intake—estimated either by spot urine sodium measurements or by 24 h urinary sodium excretion—has been associated with higher BP, an increased risk of CV events, and death [27,28]. On the other hand, a U-shaped mortality curve has emerged when sodium intake is very low (<3 gr/d) [27,29].

In CKD, hypertension is a common finding and an important CV risk factor [2]. Its prevalence skyrockets to 80–100% in CKD stages 4 and 5 [30]. On top of that, resistant hypertension is another frequent hypertension phenotype in CKD, with its prevalence being twofold higher compared to patients without CKD [31]. According to the World Health Organization (WHO) and most international guidelines and food authorities, lowering sodium intake to <2 gr/d (87 mmol) is one of the most cost-effective strategy for enhancing overall health outcomes (Table 2) [3,14,32,33]. This also applies—even more—to patients with renal disease, since CKD may reduce the ability to excrete the excess sodium. Indeed, CKD patients present a salt-sensitive BP phenotype often associated with a nondipping BP pattern that increases CV risk and accelerated disease progression [34,35,36]. In addition, sodium restriction has been reported to decrease extracellular fluid volume and improve the effectiveness of renin–angiotensin–aldosterone system (RAAS) inhibition, with some reporting a reduction in antihypertensive doses and decreasing proteinuria, thus slowing CKD progression [37,38,39]. Hence, complex pharmacological treatments alongside lifestyle changes are almost indispensable to achieve BP goals in this population, with sodium intake being a key recommendation for self-care management, among others. However, limited data are available on the “best” target for dietary sodium, and there is no established definitive target level according to eGFR and proteinuria/albuminuria levels. Although prospective randomized interventional trials assessing whether a reduction of sodium intake can slow the progression of kidney disease are lacking, with current evidence derived from observational studies, no evidence has indicated that reducing salt intake presents any significant health risk for CKD patients [40]. Only one exception has been a study in patients with type 1 diabetes and prevalent CKD, in whom a low sodium intake was associated with a higher risk of CKD [41].

In the early 2010s, a randomized trial on sodium restriction in 20 hypertensives with CKD highlighted the potential significant benefits in CKD management, as a reduction of 10/4 mmHg for systolic/diastolic BP was reported [48]. Similar BP reductions of 8/2 mmHg for systolic/diastolic BP were noticed in patients of Bangladeshi origin with CKD and BP > 130/80 mmHg who were randomized to receive a tailored low-salt diet (urinary sodium excretion fell from 260 mmol/d to 103 mmol/d at 6 months) [49]. In a 6-week randomized crossover trial in stable kidney transplant recipients on RAAS blockade, a reduction of 2 gr of sodium per day effectively reduced BP but only had a modest effect on albuminuria without affecting eGFR or causing orthostatic hypotension [50].

Adherence to a low-sodium diet seems already very challenging in hypertensive populations and even utopic in CKD patients where there are comorbidities, multiple medications, challenges in meal preparation, altered taste perception (due to uremia or medications), and socioeconomic barriers (as lower-sodium food options can be more expensive or less accessible) [5,7]. The effectiveness of a self-managed sodium restriction program in CKD patients, incorporating education, reminders about food labeling and salt content, motivational interviewing, coaching, increased awareness of the impact of sodium on BP and kidney function, and self-monitoring has also been assessed. While the interventions led to short-term reductions in sodium excretion, BP, hydration status, and proteinuria, their effects diminished over time [51,52,53].

Although practical recommendations for the general population, such as the use of salt substitutes (which often contain KCL), have been shown to reduce BP and lower CV risk [54,55,56], their use in advanced CKD or patients using a potassium-sparing diuretic raises concerns about elevated potassium levels. However, in the SSaSS study including older adults with high BP or a history of stroke, the intervention was lifesaving even in the small subgroup of CKD patients, with three averted deaths from reduction in systolic BP for each death related to hyperkalemia [56].

Overall, long-term adherence to sodium and general lifestyle interventions require strong motivation, support, and behavioral changes, which can be difficult to sustain without regular counseling or reinforcement.

## 4. Potassium Intake

The link between potassium and BP has been recognized since the 1950s, with numerous animal experiments and later human studies highlighting its beneficial role in reducing BP [57,58,59,60,61]. In recent years, several randomized control trials (RCTs) and meta-analyses have provided strong evidence that increasing potassium intake, whether through diet or supplements, effectively lowers BP [62,63,64,65]. Filippini et al. found that lower sodium and higher potassium intake may contribute to BP reduction and a decreased risk of CV disease, while also showing a U-shaped relationship in which both low and high potassium levels can negatively affect BP control [63]. This became more apparent in a study investigating the association between potassium intake and stroke risk, which found that the risk was lower with an intake up to 3500 mg/day but increased at higher levels [66]. Interestingly, the exact dose of potassium supplementation required to achieve this effect remains undetermined [62].

The favorable effects of potassium have been recognized by the international authorities, and the current global recommendations for daily potassium intake have been set to at least 3500 mg/d to reduce BP and the risk of CVD (Table 2). Surprisingly, the actual consumption in the developed world falls way below the recommendations, with significant variations among different parts of the world [67,68]. The famous Mediterranean diet may contain up to 6 g/d of potassium, whereas the DASH diet may contribute up to 4.7 g/d [69].

Recommending a high potassium intake or supplements in hypertensive CKD patients is a delicate matter due to the risk of hyperkaliemia, especially in a population largely treated with RAAS inhibitors and/or affected by concomitant diseases such as heart failure and diabetes. Most guidelines recommend limiting potassium intake for CKD patients, although the target is not consistent, and a lower limit for potassium intake ranges from 2 to 4 g/d [70,71]. However, a low-potassium diet may not be suitable for all CKD patients. Hannah et al. concluded that the DASH diet appears to be safe in patients with CKD stage 3, even when prescribed RAAS inhibitors, but careful monitoring of serum potassium may be necessary [72]. Nevertheless, studies have underlined that dietary potassium intake is not directly associated with serum potassium levels or subsequent hyperkalemia [73,74]. Bernier et al. found that, in 8,043 adults undergoing hemodialysis, dietary potassium intake was not associated with serum levels of potassium, prevalence of hyperkalemia, or all-cause mortality [75].

In addition, a potassium-rich diet typically contains plant-based foods (fruits and vegetables), meat, and fish, which also provide high amounts of fiber and alkali—both beneficial for CKD patients, as constipation and metabolic acidemia often contribute to hyperkalemia [76]. Given that the vast majority of people, including those with CKD, consume less than the recommended daily potassium intake, both potassium supplements and increased dietary intake are probably unlikely to result in excessive levels of potassium intake, at least in non-advanced CKD. Moreover, with the widespread availability of new potassium binders, increasing dietary potassium intake to obtain its undeniable benefits for BP control and CVD prevention now seems more feasible [77].

So far, evidence for potassium supplementation for CKD patients is scarce, and prospective RCTs testing the administration of potassium supplements versus placebo in this populations are lacking [40]. One small RCT compared the effect of diets containing 100 and 40 mmol potassium/day on BP in 29 adults with CKD stage 3 and concluded that a higher dietary potassium intake did not lower 24 h SBP, while office SBP reduction was of borderline statistical significance [78]. Another study examined the effect of a potassium supplement (KCl 40 mmol/day for 2 weeks) in 191 patients with CKD and found no significant changes in ambulatory or office BP [79].

As already mentioned, there is general guidance towards replacing salt with potassium, which, for example, means that, if 20% of salt is replaced by KCL, this is translated into addition of 0.45 g/d to usual intake, so it is not negligible, especially in patients with advanced CKD [80]. Salt substitutes, mainly KCL, have been associated with hyperkalemia in studies that excluded people with CKD [81], and KDIGO suggests that both the DASH diet and potassium salt substitutes may not be appropriate for advanced CKD [22]. However, a modeling study by Marklund et al. showed that nationwide implementation of potassium-enriched salt substitution in China increased lives saved, benefiting both the general and CKD populations, despite the risk of hyperkalemia [82].

In CKD patients, a continuous U-shaped relationship between serum potassium and all-cause mortality has been demonstrated [83]. Interestingly, patients with more advanced CKD stages develop protective mechanisms that enhance their tolerance to hyperkalemia [84]. The impact of dietary potassium intake on CKD prognosis remains controversial. Observational studies suggest that a high-potassium diet is associated with better renal and CV outcomes [85,86], whereas reduced dietary potassium has been associated with worse kidney outcomes in CKD patients [87]. Conversely, higher urinary potassium excretion has been associated with poorer renal outcomes in CKD patients [88]. Another noteworthy aspect is the Na/K ratio determined from frequent measurements of spot urine samples, which can provide a more informative measure of the CV risk [89]. Koo et al. in the KNOW study found that higher urinary NA/K was associated with greater risk for CKD progression [90]. Hence, Clase et al. concluded that dietary potassium restriction is not recommended as a general strategy in CKD patients [91].

## 5. Other Dietary Interventions Related to Hypertension

Calcium is another important cation, especially for patients with CKD, as many of them struggle with calcium homeostasis derangements and are frequently prescribed medications that affect its levels. The impact of dietary calcium or supplementation on BP controls remains uncertain. Hsia et al. found no effect on BP or CVD risk with increased calcium intake [92]. However, a recent meta-analysis concluded that higher calcium intake slightly reduces both SBP and DBP in normotensive individuals, particularly younger ones, suggesting a potential role in hypertension prevention [93]. Another meta-analysis reported that both calcium and magnesium were linked to significant reductions in SBP [94]. Additionally, magnesium supplementation, particularly at doses of ≥400 mg/day for ≥12 weeks, resulted in an overall decrease of SBP and DBP [95]. Patients with CKD often need to take both calcium and magnesium supplements, and, so far, there is no documented risk of exacerbating BP control attributed to them.

Although phosphorus intake is primarily discussed in the context of mineral bone disorder (CKD-MBD) and CV risk in CKD, its direct relationship with BP remains complex and inconsistent across studies. A recent systematic review of randomized trials and prospective cohort studies found no consistent association between total dietary phosphorus intake and BP [96]. Similarly, a secondary analysis of the PREMIER trial—a behavioral intervention study—showed that total phosphorus intake was not significantly associated with changes in BP over six months [97]. However, further subgroup analysis highlighted that the source of phosphorus may significantly affect outcomes: added (inorganic) phosphorus, commonly found in processed foods, was associated with statistically significant increases in both systolic and diastolic BP, whereas phosphorus from plant and animal sources did not show this association. Additionally, increased urinary phosphorus excretion was independently linked to a modest rise in diastolic BP, potentially reflecting either higher intake or altered renal handling of phosphorus.

Experimental and interventional data support the hypothesis that inorganic phosphate may contribute to elevated BP through mechanisms such as stimulation of sympathetic nervous system activity, activation of the RAAS, and altered vascular function. In one small controlled trial, healthy young adults who consumed a high-sodium phosphate diet for several weeks experienced significant increases in systolic and diastolic BP, pulse rate, and levels of PTH and FGF23 [98]. Animal studies further confirm that chronic high phosphate intake may lead to sustained hypertension, sympathetic overactivity, and vascular calcification [99,100,101]. Indeed, hyperphosphatemia contributes to vascular calcification, which increases arterial stiffness and, in turn, promotes BP increase and subsequent cardiac hypertrophy, leading potentially to a vicious cycle in CKD patients [102]. Nevertheless, hypertension may not be the primary mediator of phosphate-induced cardiac injury in the setting of CKD, as neither low- nor high-phosphate diets appear to modify elevated BP in CKD animal models. [103]

In light of all these variable findings, clinical guidelines from KDIGO and KDOQI recommend avoiding excessive phosphorus intake—particularly from inorganic additives—in patients with CKD [22,23]. While specific quantitative targets for phosphorus intake vary depending on CKD stage and serum phosphate levels, a general dietary strategy involves limiting processed foods and phosphate additives to help manage hyperphosphatemia and reduce CV risk.

Given that dietary phosphorus is closely linked to protein intake—particularly from animal-based sources—the impact of protein consumption on BP and CKD progression also warrants attention. In the context of CKD, a high protein intake is associated with metabolic acidosis—which has been linked to an increased risk of hypertension—and hyperphosphatemia, a known contributor to vascular calcification and CV morbidity [104,105,106]. Moreover, red and processed meats are typically high in sodium, which directly contributes to increased BP and worsens hypertension, making their frequent consumption particularly harmful in patients with CKD. On top of that, high protein intake can lead to glomerular hyperfiltration, accelerating CKD progression and potentially intensifying hypertension.

Nevertheless, the evidence regarding protein consumption per se and its effect on BP remains inconclusive [107]. Recent research from Carballo-Casla et al. showed that in individuals with (CKD stages 1–3) and without CKD, higher protein consumption is associated with improved survival compared to lower protein consumption [108]. Moreover, plant-based, unprocessed proteins have been shown to slow eGFR decline [22]. Current KDIGO and KDOQI guidelines do not recommend excessive protein intake >1.2 g/kg/day, especially from meat, in CKD patients due to its association with worse kidney outcomes [22,24]. Interestingly, KDOQI recommends a low-protein diet of 0.44–0.60 gr/kg/day or a very low-protein diet of 0.28–0.43 gr/kg/d supplemented with keto acid or amino acid analogs for metabolically stable adults with CKD stages 3–5, in order to reduce the risk of end-stage kidney disease (ESKD) and mortality. However, a personalized approach may be considered in CKD management, as a one-size-fits-all strategy may not suit older adults with mild or slowly progressing CKD and different comorbidities (e.g., diabetes), and the data supporting a low-protein diet were primarily gathered before the wide spread use of renoprotective agents such as RAAS inhibitors and SGLT2 inhibitors.

Although the focus on diet has been more on individual components (i.e., restricting sodium, phosphorus intake), specific dietary patterns, as underlined by several guidelines, such as the DASH and Mediterranean diets, are particularly relevant in the management of hypertension of CKD patients. The DASH diet, initially designed to lower BP in the general population, emphasizes fruits, vegetables, whole grains, low-fat dairy, and reduced sodium intake [15]. Similarly, the Mediterranean diet is rich in monounsaturated fats (primarily from olive oil), legumes, whole grains, fruits, vegetables, and fish [10]. In a recent systematic review, modified versions of the DASH and Mediterranean diets have been associated with significant reductions in systolic and diastolic BP in CKD patients; however, the follow-up period was very short [108]. In a recent study by Banerjee T et al. including 1110 CKD patients with hypertension, a low accordance to a DASH diet eating pattern was associated with a higher risk of progressing to ESKD, especially in those who were also diabetics [109]. Potassium and magnesium were strong mediators, dietary acid load and protein intake were partial mediators, and sodium (surprisingly), fiber, calcium, and cholesterol were not mediators of this association. Indeed, these diets may exert their benefits—beyond their favorable BP effect—through multiple mechanisms, including improved endothelial function, reduced inflammation and oxidative stress, decreased production of gut microbiota-derived metabolites linked to CKD and CV disease, reduction in serum phosphate levels, alleviation of metabolic acidosis, and improved lipid metabolism [110]. Although attention must be paid to potassium and phosphorus intake, particularly in advanced CKD stages, these patterns offer a comprehensive and adaptable framework for dietary management that goes beyond isolated nutrient restrictions.

Interestingly, there is emerging evidence that zinc deficiency is associated with hypertension and worse kidney and CV outcomes [111]. Studies have shown that CKD patients have lower zinc levels [112], while zinc supplementation may be have antihypertensive and renoprotective effects [113]. However, no special recommendations exist concerning the daily amount of zinc in people with hypertension or with CKD.

For all the dietary components mentioned above, there are no clear guideline recommendations due to gaps in the evidence, and recommendations from food authorities may vary significantly (Table 2).

Finally, there are studies reporting that moderate alcohol consumption is not harmful for kidney survival and may even be protective against ESRD in Chinese men [114,115]. However, heavy alcohol consumption is associated with worse kidney survival [116]. Roerecke et al. found that reducing alcohol intake lowers BP in a dose-dependent manner, and ESH 2023 recommends reduced alcohol consumption, close to abstinence [3,117]. No specific guidance is issued for CKD patients, due to lack of trials in this population.

## 6. Discussion

**Figure 1:**
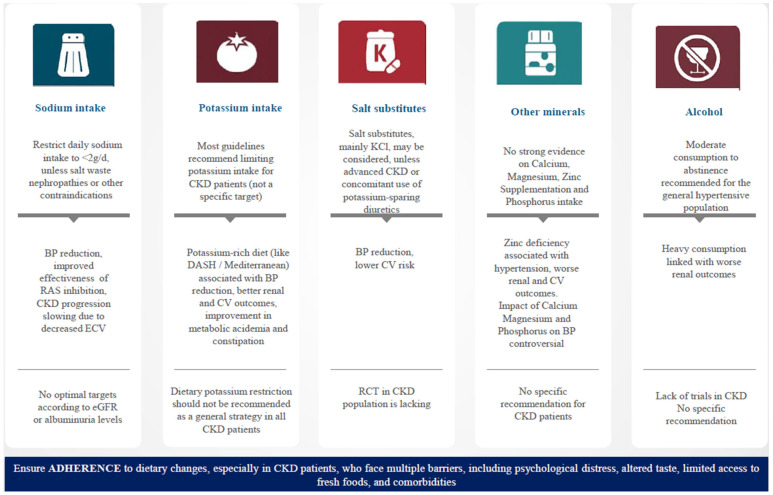
Summary of the most important dietary interventions in hypertensive patients with chronic kidney disease. BP, blood pressure; CV, cardiovascular; CKD, chronic kidney disease; RAS, renin–angiotensin system; DASH, dietary approaches to stop hypertension; RCT, randomized control trial.

The relationship between CKD and hypertension is complex; high BP can accelerate kidney damage, whereas impaired kidney function may worsen BP regulation. Several factors such as sodium, potassium, and dietary protein have a role in the pathogenesis of hypertension [118,119]. Therefore, adoption of dietary changes is an effective strategy for controlling hypertension and preserving kidney function in CKD patients. Key dietary strategies include reducing sodium intake, optimizing potassium intake, and maintaining a healthy, balanced diet (Figure 1). Additionally, focusing on healthy fats, managing fluid intake, and limiting alcohol consumption are also important. Regular monitoring by a healthcare provider or dietitian is essential to ensure a proper balance of nutrients, especially in more advanced stages of CKD.

Despite the established role of dietary interventions in managing hypertension among CKD patients, current recommendations vary across international and regional guidelines. In this review, we examined and compared dietary guidance from several major bodies such as KDIGO, the AHA, the ADA, and the ESH, among others. Importantly, we also included recommendations from overseas and non-Western guidelines to capture a broader, more globally representative view of dietary recommendations across diverse healthcare settings. By doing so, we underscored both shared principles—such as the consistent emphasis on sodium reduction—and notable discrepancies, particularly in potassium recommendations, which are often stage-specific and dependent on individual risk factors such as hyperkalemia or, in some cases, not explicitly addressed at all.

Beyond regional differences, it is also important to acknowledge how guidance has evolved over time, reflecting new evidence and changing clinical priorities. Substances once considered uniformly harmful are now being re-evaluated in the context of individual patient profiles and CKD stages. For example, potassium—traditionally restricted in advanced CKD due to the risk of hyperkalemia—is now being cautiously reconsidered, particularly in early-stage CKD or in patients with preserved urinary excretion, where increased intake may offer cardiovascular benefits. Similarly, although protein intake per se exhibits a modest effect on BP, a high-protein diet can adversely affect kidney function by inducing glomerular hyperfiltration and metabolic acidosis, thereby contributing indirectly to long-term BP elevation [120,121,122]. Indeed, the role of dietary protein is no longer viewed in binary terms of restriction vs. excess; instead, recent guidelines emphasize tailored intake based on disease progression, nutritional status, and metabolic needs. These shifts underscore the importance of individualized care plans that incorporate the latest clinical evidence.

Despite the dynamic nature of dietary management in CKD, sodium intake remains a central focus due to its strong association with BP regulation in CKD. Recognizing sodium as a key factor in the onset and management of hypertension has led to multiple efforts targeting a reduction of daily sodium intake, but these have yielded disappointing results. Recent evidence suggests that, rather than solely focusing on restraining sodium consumption, targeting the increase in potassium consumption may be a more successful strategy against the hypertension epidemic. Substituting NaCl with KCl seems to be a relatively safe and feasible approach for both the general population and patients with non-advanced CKD. Hence, shifting towards and promoting the adoption of a more plant-based diet rich in potassium, lower in meat, and containing fewer preservatives—rather than focusing solely on sodium restriction, is a potential valuable intervention worthy on time and resource investment, as it promotes overall health. Finally, as with all treatments, compliance is a major issue. Most people can follow dietary modifications, but the situation worsens when guidelines are unclear or the goals are unrealistic, leading to adherence being sustained for only a limited period. Healthcare professionals play a key role in providing ongoing guidance, encouragement, and tailored intervention to ensure long-term success, as adding more and more pharmacological treatments without first achieving the goals of lifestyle interventions is not only insufficient but harmful too. Also, the valuable role of renal dietitians has been recently highlighted by the 2024 Kidney Disease: Improving Global Outcome (KDIGO) Guidelines [22].

In conclusion, although international guidelines and food authorities do not have major differences in aspects such as salt restriction, other areas that may be improved are the recommendations concerning potassium intake, the role of non-pharmacological intervention, and the support provided through advice and education from a renal dietitian. If all these become clearer, then both medical professionals and patients will become less confused and more convinced about which strategy to follow. Towards this direction, new randomized prospective studies that will cover this gap and provide concrete evidence regarding the interventions that should be followed, especially in patients with CKD, are needed.

## Figures and Tables

**Table 2:** Dietary recommendations for daily intake in the general population, according to food authorities [32,42,43,44,45,46,47].

Food Authority	Sodium/Na (Salt/NaCl)	Potassium	Calcium	Magnesium	Protein	Zinc	Phosphorus
FAO/ WHO	<2000 mg Na (5 g)	3510 mg	1000 mg	M: 260 mg W: 220 mg	0.75 g/kg	M: 14 mg W: 10 mg	700 mg
EFSA	<5 g salt	3500 mg	1000 mg	M: 375 mg W: 310 mg	0.83 g/kg	M: 11 mg W: 8 mg	700 mg
FDA	2300 mg Na	4700 mg	1300 mg	420 mg	50 g	11 mg	1250 mg
NHS	2400 mg Na (6 g)	3500 mg	700 mg	M: 300 mg W: 270 mg	-	M: 9.5 mg W: 7 mg	550 mg
FSSAI	1900–2100 mg Na	M: 3750 mg W: 3225 mg	600 mg	M: 340 mg W: 310 mg	M: 60g W: 55g	M: 12 mg W: 10 mg	600 mg
Health Canada	<2300 mg Na	M: 3400 mg W: 2800 mg	1000 mg	M: 400 mg W: 320 mg	M: 56g W: 46g	M: 11 mg W: 8 mg	700 mg
Food Standards Australia and New Zealand	<2000 mg Na	M: 3800 mg W: 2800 mg	1000 mg	M: 420 mg W: 320 mg	M: 0.84 g/kg (64 g) W: 0.75 g/kg (46 g)	M: 14 mg W:8 mg	1000 mg

FAO/WHO, Food and Agriculture Organization (FAO) and World Health Organization (WHO); EFSA, European Food Safety Authority; FDA, U.S. Food and Drug Administration; NHS, National Health Service; FSSAI, Food Safety and Standards Authority of India; M, Men; W, Women.

## Data Availability

No new data were created or analyzed in this study. Data sharing is not applicable to this article.

## Abbreviations

The following abbreviations are used in this manuscript:

CKD	Chronic Kidney Disease
BP	Blood Pressure
CV	Cardio Vascular
DASH	Dietary Approaches to Stop Hypertension
ESH	European Society of Hypertension
ESC	European Society of Cardiology
KDIGO	Kidney Disease Improving Global Outcomes
RAAS	Renin-Angiotensin-Aldosterone System
KDOQI	Kidney Disease Outcomes Quality Initiative
eGFR	Estimated Glomerular Filtration Rate
WHO	World Health Organization
RCT	Randomized Control Trial

## References

[1] Akbari S., Ten Eyck P., Wendt L., Yamada M., Boucher R., Beddhu S., Jalal D.I. (2024). Trends of Blood Pressure Control in Chronic Kidney Disease Among US Adults: Findings from NHANES 2011 to 2020. J. Am. Heart Assoc..

[2] Burnier M., Damianaki A. (2023). Hypertension as Cardiovascular Risk Factor in Chronic Kidney Disease. Circ. Res..

[3] Mancia G., Kreutz R., Brunstrom M., Burnier M., Grassi G., Januszewicz A., Muiesan M.L., Tsioufis K., Agabiti-Rosei E., Algharably E.A.E. (2023). 2023 ESH Guidelines for the management of arterial hypertension the Task Force for the management of arterial hypertension of the European Society of Hypertension: Endorsed by the International Society of Hypertension (ISH) and the European Renal Association (ERA). J. Hypertens..

[4] Cheung A.K., Chang T.I., Cushman W.C., Furth S.L., Hou F.F., Ix J.H., Knoll G.A., Muntner P., Pecoits-Filho R., Sarnak M.J. (2021). KDIGO 2021 Clinical Practice Guideline for the Management of Blood Pressure in Chronic Kidney Disease. Kidney Int..

[5] Trigueros-Flores X.B., Luna-Hernandez G., Santos-Lopez M.F., Perez-Galvan L., Flores-Camacho K.J., Diaz-Canchola L.M., Cueto-Manzano A.M., Chavez-Chavez H.E., Cerrillos-Gutierrez J.I., Rojas-Campos E. (2025). Barriers and Facilitators to Adherence to a Healthy Diet Across the Spectrum of Chronic Kidney Disease. Patient Prefer. Adherence.

[6] Cardol C.K., Boslooper-Meulenbelt K., van Middendorp H., Meuleman Y., Evers A.W.M., van Dijk S. (2022). Psychosocial barriers and facilitators for adherence to a healthy lifestyle among patients with chronic kidney disease: A focus group study. BMC Nephrol..

[7] Meuleman Y., Ten Brinke L., Kwakernaak A.J., Vogt L., Rotmans J.I., Bos W.J., van der Boog P.J., Navis G., van Montfrans G.A., Hoekstra T. (2015). Perceived Barriers and Support Strategies for Reducing Sodium Intake in Patients with Chronic Kidney Disease: A Qualitative Study. Int. J. Behav. Med..

[8] Tyson C.C., Svetkey L.P., Lin P.H., Granados I., Kennedy D., Dunbar K.T., Redd C., Bennett G., Boulware L.E., Fish L.J. (2023). Self-Perceived Barriers and Facilitators to Dietary Approaches to Stop Hypertension Diet Adherence Among Black Americans with Chronic Kidney Disease: A Qualitative Study. J. Ren. Nutr..

[9] Filippou C.D., Tsioufis C.P., Thomopoulos C.G., Mihas C.C., Dimitriadis K.S., Sotiropoulou L.I., Chrysochoou C.A., Nihoyannopoulos P.I., Tousoulis D.M. (2020). Dietary approaches to stop hypertension (DASH) diet and blood pressure reduction in adults with and without hypertension: A systematic review and meta-analysis of randomized controlled trials. Adv. Nutr..

[10] Filippou C.D., Thomopoulos C.G., Kouremeti M.M., Sotiropoulou L.I., Nihoyannopoulos P.I., Tousoulis D.M., Tsioufis C.P. (2021). Mediterranean diet and blood pressure reduction in adults with and without hypertension: A systematic review and meta-analysis of randomized controlled trials. Clin. Nutr..

[11] Fu J., Liu Y., Zhang L., Zhou L., Li D., Quan H., Zhu L., Hu F., Li X., Meng S. (2020). Nonpharmacologic interventions for reducing blood pressure in adults with prehypertension to established hypertension. J. Am. Heart Assoc..

[12] He F.J., Li J., Macgregor G.A. (2013). Effect of longer-term modest salt reduction on blood pressure. Cochrane Database Syst. Rev..

[13] McEvoy J.W., McCarthy C.P., Bruno R.M., Brouwers S., Canavan M.D., Ceconi C., Christodorescu R.M., Daskalopoulou S.S., Ferro C.J., Gerdts E. (2024). 2024 ESC Guidelines for the management of elevated blood pressure and hypertension. Eur. Heart J..

[14] Whelton P.K., Carey R.M., Aronow W.S., Casey D.E., Collins K.J., Dennison Himmelfarb C., DePalma S.M., Gidding S., Jamerson K.A., Jones D.W. (2018). 2017 ACC/AHA/AAPA/ABC/ACPM/AGS/APhA/ASH/ASPC/NMA/PCNA Guideline for the Prevention, Detection, Evaluation, and Management of High Blood Pressure in Adults: Executive Summary: A Report of the American College of Cardiology/American Heart Association Task Force on Clinical Practice Guidelines. Hypertension.

[15] Appel L.J., Moore T.J., Obarzanek E., Vollmer W.M., Svetkey L.P., Sacks F.M., Bray G.A., Vogt T.M., Cutler J.A., Windhauser M.M. (1997). A clinical trial of the effects of dietary patterns on blood pressure. DASH Collaborative Research Group. N. Engl. J. Med..

[16] Whelton P.K., He J., Cutler J.A., Brancati F.L., Appel L.J., Follmann D., Klag M.J. (1997). Effects of oral potassium on blood pressure. Meta-analysis of randomized controlled clinical trials. JAMA.

[17] Aburto N.J., Hanson S., Gutierrez H., Hooper L., Elliott P., Cappuccio F.P. (2013). Effect of increased potassium intake on cardiovascular risk factors and disease: Systematic review and meta-analyses. BMJ.

[18] Graudal N.A., Hubeck-Graudal T., Jurgens G. (2011). Effects of low sodium diet versus high sodium diet on blood pressure, renin, aldosterone, catecholamines, cholesterol, and triglyceride. Cochrane Database Syst. Rev..

[19] Kim K.I., Ihm S.H., Kim G.H., Kim H.C., Kim J.H., Lee H.Y., Lee J.H., Park J.M., Park S., Pyun W.B. (2019). 2018 Korean society of hypertension guidelines for the management of hypertension: Part III-hypertension in special situations. Clin. Hypertens..

[20] Kim H.L., Lee E.M., Ahn S.Y., Kim K.I., Kim H.C., Kim J.H., Lee H.Y., Lee J.H., Park J.M., Cho E.J. (2023). The 2022 focused update of the 2018 Korean Hypertension Society Guidelines for the management of hypertension. Clin. Hypertens..

[21] Camafort M., Kasiakogias A., Agabiti-Rosei E., Masi S., Iliakis P., Benetos A., Jeong J.O., Lee H.Y., Muiesan M.L., Sudano I. (2025). Hypertensive heart disease in older patients: Considerations for clinical practice. Eur. J. Intern. Med..

[22] Stevens P.E., Ahmed S.B., Carrero J.J., Foster B., Francis A., Hall R.K., Herrington W.G., Hill G., Inker L.A., Kazancıoğlu R. (2024). KDIGO 2024 clinical practice guideline for the evaluation and management of chronic kidney disease. Kidney Int..

[23] Navaneethan S.D., Bansal N., Cavanaugh K.L., Chang A., Crowley S., Delgado C., Estrella M.M., Ghossein C., Ikizler T.A., Koncicki H. (2025). KDOQI US Commentary on the KDIGO 2024 Clinical Practice Guideline for the Evaluation and Management of CKD. Am. J. Kidney Dis..

[24] Ikizler T.A., Burrowes J.D., Byham-Gray L.D., Campbell K.L., Carrero J.-J., Chan W., Fouque D., Friedman A.N., Ghaddar S., Goldstein-Fuchs D.J. (2020). KDOQI Clinical Practice Guideline for Nutrition in CKD: 2020 Update. Am. J. Kidney Dis..

[25] Pollock C., Moon J.-y., Ngoc Ha L.P., Gojaseni P., Ching C.H., Gomez L., Chan T.M., Wu M.-J., Yeo S.C., Nugroho P. (2024). Framework of Guidelines for Management of CKD in Asia. Kidney Int. Rep..

[26] American Diabetes Association Professional Practice Committee (2024). 11. Chronic Kidney Disease and Risk Management: Standards of Care in Diabetes—2025. Diabetes Care.

[27] Mente A., O’Donnell M., Rangarajan S., Dagenais G., Lear S., McQueen M., Diaz R., Avezum A., Lopez-Jaramillo P., Lanas F. (2016). Associations of urinary sodium excretion with cardiovascular events in individuals with and without hypertension: A pooled analysis of data from four studies. Lancet.

[28] Huang L., Trieu K., Yoshimura S., Neal B., Woodward M., Campbell N.R.C., Li Q., Lackland D.T., Leung A.A., Anderson C.A.M. (2020). Effect of dose and duration of reduction in dietary sodium on blood pressure levels: Systematic review and meta-analysis of randomised trials. BMJ.

[29] O’Donnell M., Mente A., Rangarajan S., McQueen M.J., Wang X., Liu L., Yan H., Lee S.F., Mony P., Devanath A. (2014). Urinary sodium and potassium excretion, mortality, and cardiovascular events. N. Engl. J. Med..

[30] Muntner P., Anderson A., Charleston J., Chen Z., Ford V., Makos G., O’Connor A., Perumal K., Rahman M., Steigerwalt S. (2010). Hypertension awareness, treatment, and control in adults with CKD: Results from the Chronic Renal Insufficiency Cohort (CRIC) Study. Am. J. Kidney Dis..

[31] Tanner R.M., Calhoun D.A., Bell E.K., Bowling C.B., Gutierrez O.M., Irvin M.R., Lackland D.T., Oparil S., Warnock D., Muntner P. (2013). Prevalence of apparent treatment-resistant hypertension among individuals with CKD. Clin. J. Am. Soc. Nephrol..

[32] WHO (2012). Guideline: Sodium Intake for Adults and Children.

[33] Unger T., Borghi C., Charchar F., Khan N.A., Poulter N.R., Prabhakaran D., Ramirez A., Schlaich M., Stergiou G.S., Tomaszewski M. (2020). 2020 International Society of Hypertension Global Hypertension Practice Guidelines. Hypertension.

[34] Burnier M., Coltamai L., Maillard M., Bochud M. (2007). Renal sodium handling and nighttime blood pressure. Semin. Nephrol..

[35] Rust P., Ekmekcioglu C., Islam M.S. (2017). Impact of Salt Intake on the Pathogenesis and Treatment of Hypertension. Hypertension: From Basic Research to Clinical Practice.

[36] Cianciaruso B., Bellizzi V., Minutolo R., Colucci G., Bisesti V., Russo D., Conte G., De Nicola L. (1996). Renal adaptation to dietary sodium restriction in moderate renal failure resulting from chronic glomerular disease. J. Am. Soc. Nephrol..

[37] Guyton A.C. (1992). Kidneys and fluids in pressure regulation. Small volume but large pressure changes. Hypertension.

[38] Borrelli S., Provenzano M., Gagliardi I., Michael A., Liberti M.E., De Nicola L., Conte G., Garofalo C., Andreucci M. (2020). Sodium Intake and Chronic Kidney Disease. Int. J. Mol. Sci..

[39] McMahon E.J., Campbell K.L., Bauer J.D., Mudge D.W., Kelly J.T. (2021). Altered dietary salt intake for people with chronic kidney disease. Cochrane Database Syst. Rev..

[40] Burnier M. (2021). Sodium Intake and Progression of Chronic Kidney Disease-Has the Time Finally Come to Do the Impossible: A Prospective Randomized Controlled Trial?.

[41] Thomas M.C., Moran J., Forsblom C., Harjutsalo V., Thorn L., Ahola A., Waden J., Tolonen N., Saraheimo M., Gordin D. (2011). The association between dietary sodium intake, ESRD, and all-cause mortality in patients with type 1 diabetes. Diabetes Care.

[42] Turck D., Bresson J.-L., Burlingame B., Dean T., Fairweather-Tait S., Heinonen M., Hirsch-Ernst K.I., Mangelsdorf I., McArdle H., EFSA Panel on Dietetic Products, Nutrition and Allergies (NDA) (2016). Dietary reference values for potassium. EFSA J..

[43] Food Safety and Standards Authority of India (2011). Nutritional Standards and Regulations.

[44] Food Standards Australia New Zealand (FSANZ) (2022). Nutrient Reference Values for Australia and New Zealand—Potassium.

[45] U.S. Food and Drug Administration (2024). Daily Value on the Nutrition and Supplement Facts Labels.

[46] Government of Canada Health Canada (2023). Dietary Reference Intakes: Potassium.

[47] National Health Service (2020). Vitamins and Minerals: Potassium.

[48] McMahon E.J., Bauer J.D., Hawley C.M., Isbel N.M., Stowasser M., Johnson D.W., Campbell K.L. (2013). A randomized trial of dietary sodium restriction in CKD. J. Am. Soc. Nephrol..

[49] de Brito-Ashurst I., Perry L., Sanders T.A., Thomas J.E., Dobbie H., Varagunam M., Yaqoob M.M. (2013). The role of salt intake and salt sensitivity in the management of hypertension in South Asian people with chronic kidney disease: A randomised controlled trial. Heart.

[50] de Vries L.V., Dobrowolski L.C., van den Bosch J.J., Riphagen I.J., Krediet C.T., Bemelman F.J., Bakker S.J., Navis G. (2016). Effects of Dietary Sodium Restriction in Kidney Transplant Recipients Treated with Renin-Angiotensin-Aldosterone System Blockade: A Randomized Clinical Trial. Am. J. Kidney Dis..

[51] Meuleman Y., Hoekstra T., Dekker F.W., Navis G., Vogt L., van der Boog P.J.M., Bos W.J.W., van Montfrans G.A., van Dijk S., ESMO Study Group (2017). Sodium Restriction in Patients with CKD: A Randomized Controlled Trial of Self-management Support. Am. J. Kidney Dis..

[52] Saran R., Padilla R.L., Gillespie B.W., Heung M., Hummel S.L., Derebail V.K., Pitt B., Levin N.W., Zhu F., Abbas S.R. (2017). A Randomized Crossover Trial of Dietary Sodium Restriction in Stage 3-4 CKD. Clin. J. Am. Soc. Nephrol..

[53] O’Callaghan C.A., Camidge C., Thomas R., Reschen M.E., Maycock A.J., Lasserson D.S., Fox R.A., Thomas N.P.B., Shine B., James T. (2023). Evaluation of a Simple Low-cost Intervention to Empower People with CKD to Reduce Their Dietary Salt Intake: OxCKD1, a Multicenter Randomized Controlled Trial. Kidney360.

[54] Bernabe-Ortiz A., Sal Y.R.V.G., Ponce-Lucero V., Cardenas M.K., Carrillo-Larco R.M., Diez-Canseco F., Pesantes M.A., Sacksteder K.A., Gilman R.H., Miranda J.J. (2020). Effect of salt substitution on community-wide blood pressure and hypertension incidence. Nat. Med..

[55] Hernandez A.V., Emonds E.E., Chen B.A., Zavala-Loayza A.J., Thota P., Pasupuleti V., Roman Y.M., Bernabe-Ortiz A., Miranda J.J. (2019). Effect of low-sodium salt substitutes on blood pressure, detected hypertension, stroke and mortality. Heart.

[56] Neal B., Wu Y., Feng X., Zhang R., Zhang Y., Shi J., Zhang J., Tian M., Huang L., Li Z. (2021). Effect of Salt Substitution on Cardiovascular Events and Death. N. Engl. J. Med..

[57] Meneely G.R., Tucker R.G., Darby W.J., Auerbach S.H. (1953). Chronic sodium chloride toxicity: Hypertension, renal and vascular lesions. Ann. Intern. Med..

[58] Tobian L., Lange J., Ulm K., Wold L., Iwai J. (1985). Potassium reduces cerebral hemorrhage and death rate in hypertensive rats, even when blood pressure is not lowered. Hypertension.

[59] Tobian L. (1997). Dietary sodium chloride and potassium have effects on the pathophysiology of hypertension in humans and animals. Am. J. Clin. Nutr..

[60] Khaw K.T., Thom S. (1982). Randomised double-blind cross-over trial of potassium on blood-pressure in normal subjects. Lancet.

[61] Macgregor G., Markandu N., Smith S., Banks R., Sagnella G. (1982). Moderate potassium supplementation in essential hypertension. Lancet.

[62] Binia A., Jaeger J., Hu Y., Singh A., Zimmermann D. (2015). Daily potassium intake and sodium-to-potassium ratio in the reduction of blood pressure: A meta-analysis of randomized controlled trials. J. Hypertens..

[63] Filippini T., Naska A., Kasdagli M.I., Torres D., Lopes C., Carvalho C., Moreira P., Malavolti M., Orsini N., Whelton P.K. (2020). Potassium intake and blood pressure: A dose-response meta-analysis of randomized controlled trials. J. Am. Heart Assoc..

[64] Poorolajal J., Zeraati F., Soltanian A.R., Sheikh V., Hooshmand E., Maleki A. (2017). Oral potassium supplementation for management of essential hypertension: A meta-analysis of randomized controlled trials. PLoS ONE.

[65] Yin X., Rodgers A., Perkovic A., Huang L., Li K.-C., Yu J., Wu Y., Wu J., Marklund M., Huffman M.D. (2022). Effects of salt substitutes on clinical outcomes: A systematic review and meta-analysis. Heart.

[66] Vinceti M., Filippini T., Crippa A., de Sesmaisons A., Wise L.A., Orsini N. (2016). Meta-analysis of potassium intake and the risk of stroke. J. Am. Heart Assoc..

[67] Reddin C., Ferguson J., Murphy R., Clarke A., Judge C., Griffith V., Alvarez A., Smyth A., Mente A., Yusuf S. (2023). Global mean potassium intake: A systematic review and Bayesian meta-analysis. Eur. J. Nutr..

[68] Sebastian A., Cordain L., Frassetto L., Banerjee T., Morris R.C. (2018). Postulating the major environmental condition resulting in the expression of essential hypertension and its associated cardiovascular diseases: Dietary imprudence in daily selection of foods in respect of their potassium and sodium content resulting in oxidative stress-induced dysfunction of the vascular endothelium, vascular smooth muscle, and perivascular tissues. Med. Hypotheses.

[69] Tyson C.C., Nwankwo C., Lin P.-H., Svetkey L.P. (2012). The Dietary Approaches to Stop Hypertension (DASH) eating pattern in special populations. Curr. Hypertens. Rep..

[70] Kalantar-Zadeh K., Fouque D. (2017). Nutritional management of chronic kidney disease. N. Engl. J. Med..

[71] Picard K., Silva M.I.B., Mager D., Richard C. (2020). Dietary potassium intake and risk of chronic kidney disease progression in predialysis patients with chronic kidney disease: A systematic review. Adv. Nutr..

[72] Hannah J., Wells L., Jones C. (2018). The feasibility of using the Dietary Approaches to Stop Hypertension (DASH) diet in people with chronic kidney disease and hypertension. J. Clin. Nephrol. Kidney Dis..

[73] Morimoto N., Shioji S., Akagi Y., Fujiki T., Mandai S., Ando F., Mori T., Susa K., Naito S., Sohara E. (2024). Associations between dietary potassium intake from different food sources and hyperkalemia in patients with chronic kidney disease. J. Ren. Nutr..

[74] Ogata S., Akashi Y., Kato S., Oka Y., Suda A., Yoshizaki S., Maeda Y., Nishimura K., Maeda K., Nakai S. (2023). Association between dietary potassium intake estimated from multiple 24-hour urine collections and serum potassium in patients with CKD. Kidney Int. Rep..

[75] Bernier-Jean A., Wong G., Saglimbene V., Ruospo M., Palmer S.C., Natale P., Garcia-Larsen V., Johnson D.W., Tonelli M., Hegbrant J. (2021). Dietary potassium intake and all-cause mortality in adults treated with hemodialysis. Clin. J. Am. Soc. Nephrol..

[76] De Nicola L., Garofalo C., Borrelli S., Minutolo R. (2022). Recommendations on nutritional intake of potassium in CKD: It’s now time to be more flexible!. Kidney Int..

[77] Avesani C.M., Heimbürger O., Rubin C., Sallstrom T., Fáxen-Irving G., Lindholm B., Stenvinkel P. (2024). Plant-based diet in hyperkalemic chronic kidney disease patients receiving sodium zirconium cyclosilicate: A feasibility clinical trial. Am. J. Clin. Nutr..

[78] Turban S., Juraschek S.P., Miller III E.R., Anderson C.A., White K., Charleston J., Appel L.J. (2021). Randomized trial on the effects of dietary potassium on blood pressure and serum potassium levels in adults with chronic kidney disease. Nutrients.

[79] Gritter M., Wouda R.D., Yeung S.M., Wieërs M.L., Geurts F., De Ridder M.A., Ramakers C.R., Vogt L., De Borst M.H., Rotmans J.I. (2022). Effects of short-term potassium chloride supplementation in patients with CKD. J. Am. Soc. Nephrol..

[80] Van Buren L., Dötsch-Klerk M., Seewi G., Newson R.S. (2016). Dietary impact of adding potassium chloride to foods as a sodium reduction technique. Nutrients.

[81] Cappuccio F.P., Buchanan L.A., Ji C., Siani A., Miller M.A. (2016). Systematic review and meta-analysis of randomised controlled trials on the effects of potassium supplements on serum potassium and creatinine. BMJ Open.

[82] Marklund M., Singh G., Greer R., Cudhea F., Matsushita K., Micha R., Brady T., Zhao D., Huang L., Tian M. (2020). Estimated population wide benefits and risks in China of lowering sodium through potassium enriched salt substitution: Modelling study. BMJ.

[83] Kovesdy C.P., Matsushita K., Sang Y., Brunskill N.J., Carrero J.J., Chodick G., Hasegawa T., Heerspink H.L., Hirayama A., Landman G.W.D. (2018). Serum potassium and adverse outcomes across the range of kidney function: A CKD Prognosis Consortium meta-analysis. Eur. Heart J..

[84] Gasparini A., Evans M., Barany P., Xu H., Jernberg T., Arnlov J., Lund L.H., Carrero J.J. (2019). Plasma potassium ranges associated with mortality across stages of chronic kidney disease: The Stockholm CREAtinine Measurements (SCREAM) project. Nephrol. Dial. Transplant..

[85] Smyth A., Dunkler D., Gao P., Teo K.K., Yusuf S., O’Donnell M.J., Mann J.F., Clase C.M., Ontarget and Transcend Investigators (2014). The relationship between estimated sodium and potassium excretion and subsequent renal outcomes. Kidney Int..

[86] Araki S., Haneda M., Koya D., Kondo K., Tanaka S., Arima H., Kume S., Nakazawa J., Chin-Kanasaki M., Ugi S. (2015). Urinary Potassium Excretion and Renal and Cardiovascular Complications in Patients with Type 2 Diabetes and Normal Renal Function. Clin. J. Am. Soc. Nephrol..

[87] de Rooij E.N., de Fijter J.W., Le Cessie S., Hoorn E.J., Jager K.J., Chesnaye N.C., Evans M., Windahl K., Caskey F.J., Torino C. (2023). Serum potassium and risk of death or kidney replacement therapy in older people with CKD stages 4–5: Eight-year follow-up. Am. J. Kidney Dis..

[88] He J., Mills K.T., Appel L.J., Yang W., Chen J., Lee B.T., Rosas S.E., Porter A., Makos G., Weir M.R. (2016). Urinary Sodium and Potassium Excretion and CKD Progression. J. Am. Soc. Nephrol..

[89] Iwahori T., Miura K., Ueshima H. (2017). Time to Consider Use of the Sodium-to-Potassium Ratio for Practical Sodium Reduction and Potassium Increase. Nutrients.

[90] Koo H., Hwang S., Kim T.H., Kang S.W., Oh K.-H., Ahn C., Kim Y.H. (2018). The ratio of urinary sodium and potassium and chronic kidney disease progression: Results from the KoreaN Cohort Study for Outcomes in Patients with Chronic Kidney Disease (KNOW-CKD). Medicine.

[91] Clase C.M., Carrero J.-J., Ellison D.H., Grams M.E., Hemmelgarn B.R., Jardine M.J., Kovesdy C.P., Kline G.A., Lindner G., Obrador G.T. (2020). Potassium homeostasis and management of dyskalemia in kidney diseases: Conclusions from a Kidney Disease: Improving Global Outcomes (KDIGO) Controversies Conference. Kidney Int..

[92] Hsia J., Heiss G., Ren H., Allison M., Dolan N.C., Greenland P., Heckbert S.R., Johnson K.C., Manson J.E., Sidney S. (2007). Calcium/vitamin D supplementation and cardiovascular events. Circulation.

[93] Cormick G., Ciapponi A., Cafferata M.L., Belizán J.M. (2015). Calcium supplementation for prevention of primary hypertension. Cochrane Database Syst. Rev..

[94] Behers B.J., Melchor J., Behers B.M., Meng Z., Swanson P.J., Paterson H.I., Mendez Araque S.J., Davis J.L., Gerhold C.J., Shah R.S. (2023). Vitamins and Minerals for Blood Pressure Reduction in the General, Normotensive Population: A Systematic Review and Meta-Analysis of Six Supplements. Nutrients.

[95] Alharran A.M., Alzayed M.M., Jamilian P., Prabahar K., Kamal A.H., Alotaibi M.N., Elshaer O.E., Alhatm M., Masmoum M.D., Hernández-Wolters B. (2024). Impact of Magnesium Supplementation on Blood Pressure: An Umbrella Meta-Analysis of Randomized Controlled Trials. Curr. Ther. Res. Clin. Exp..

[96] McClure S.T., Rebholz C.M., Medabalimi S., Hu E.A., Xu Z., Selvin E., Appel L.J. (2019). Dietary phosphorus intake and blood pressure in adults: A systematic review of randomized trials and prospective observational studies. Am. J. Clin. Nutr..

[97] McClure S.T., Rebholz C.M., Mitchell D.C., Selvin E., Appel L.J. (2020). The association of dietary phosphorus with blood pressure: Results from a secondary analysis of the PREMIER trial. J. Hum. Hypertens..

[98] Mohammad J., Scanni R., Bestmann L., Hulter H.N., Krapf R. (2018). A Controlled Increase in Dietary Phosphate Elevates BP in Healthy Human Subjects. J. Am. Soc. Nephrol..

[99] Latic N., Peitzsch M., Zupcic A., Pietzsch J., Erben R.G. (2022). Long-Term Excessive Dietary Phosphate Intake Increases Arterial Blood Pressure, Activates the Renin-Angiotensin-Aldosterone System, and Stimulates Sympathetic Tone in Mice. Biomedicines.

[100] Mizuno M., Mitchell J.H., Crawford S., Huang C.-L., Maalouf N., Hu M.-C., Moe O.W., Smith S.A., Vongpatanasin W. (2016). High dietary phosphate intake induces hypertension and augments exercise pressor reflex function in rats. Am. J. Physiol. -Regul. Integr. Comp. Physiol..

[101] Bozic M., Panizo S., Sevilla M.A., Riera M., Soler M.J., Pascual J., Lopez I., Freixenet M., Fernandez E., Valdivielso J.M. (2014). High phosphate diet increases arterial blood pressure via a parathyroid hormone mediated increase of renin. J. Hypertens..

[102] Campos I., Faul C. (2025). Elevated phosphate levels in CKD—A direct threat for the heart. Nephrol. Dial. Transplant..

[103] Neves K.R., Graciolli F.G., dos Reis L.M., Pasqualucci C.A., Moysés R.M., Jorgetti V. (2004). Adverse effects of hyperphosphatemia on myocardial hypertrophy, renal function, and bone in rats with renal failure. Kidney Int..

[104] Nasrallah M.M., El-Shehaby A.R., Salem M.M., Osman N.A., El Sheikh E., Sharaf El Din U.A. (2010). Fibroblast growth factor-23 (FGF-23) is independently correlated to aortic calcification in haemodialysis patients. Nephrol. Dial. Transplant..

[105] Chang A.R., Lazo M., Appel L.J., Gutierrez O.M., Grams M.E. (2014). High dietary phosphorus intake is associated with all-cause mortality: Results from NHANES III. Am. J. Clin. Nutr..

[106] Kalantar-Zadeh K., Joshi S., Schlueter R., Cooke J., Brown-Tortorici A., Donnelly M., Schulman S., Lau W.L., Rhee C.M., Streja E. (2020). Plant-Dominant Low-Protein Diet for Conservative Management of Chronic Kidney Disease. Nutrients.

[107] Boeing H., Amini A.M., Haardt J., Schmidt A., Bischoff-Ferrari H.A., Buyken A.E., Egert S., Ellinger S., Kroke A., Lorkowski S. (2024). Dietary protein and blood pressure: An umbrella review of systematic reviews and evaluation of the evidence. Eur. J. Nutr..

[108] Carballo-Casla A., Avesani C.M., Beridze G., Ortolá R., García-Esquinas E., Lopez-Garcia E., Dai L., Dunk M.M., Stenvinkel P., Lindholm B. (2024). Protein Intake and Mortality in Older Adults with Chronic Kidney Disease. JAMA Netw. Open.

[109] Palmer S.C., Maggo J.K., Campbell K.L., Craig J.C., Johnson D.W., Sutanto B., Ruospo M., Tong A., Strippoli G.F. (2017). Dietary interventions for adults with chronic kidney disease. Cochrane Database Syst. Rev..

[110] Banerjee T., Crews D.C., Tuot D.S., Pavkov M.E., Burrows N.R., Stack A.G., Saran R., Bragg-Gresham J., Powe N.R., Centers for Disease C. (2019). Poor accordance to a DASH dietary pattern is associated with higher risk of ESRD among adults with moderate chronic kidney disease and hypertension. Kidney Int..

[111] Apetrii M., Timofte D., Voroneanu L., Covic A. (2021). Nutrition in Chronic Kidney Disease-The Role of Proteins and Specific Diets. Nutrients.

[112] Ume A.C., Wenegieme T.Y., Adams D.N., Adesina S.E., Williams C.R. (2023). Zinc Deficiency: A Potential Hidden Driver of the Detrimental Cycle of Chronic Kidney Disease and Hypertension. Kidney360.

[113] Damianaki K., Lourenco J.M., Braconnier P., Ghobril J.P., Devuyst O., Burnier M., Lenglet S., Augsburger M., Thomas A., Pruijm M. (2020). Renal handling of zinc in chronic kidney disease patients and the role of circulating zinc levels in renal function decline. Nephrol. Dial. Transplant..

[114] Li M.S., Adesina S.E., Ellis C.L., Gooch J.L., Hoover R.S., Williams C.R. (2017). NADPH oxidase-2 mediates zinc deficiency-induced oxidative stress and kidney damage. Am. J. Physiol. Cell Physiol..

[115] Lai Y.J., Chen Y.Y., Lin Y.K., Chen C.C., Yen Y.F., Deng C.Y. (2019). Alcohol Consumption and Risk of Chronic Kidney Disease: A Nationwide Observational Cohort Study. Nutrients.

[116] Reynolds K., Gu D., Chen J., Tang X., Yau C.L., Yu L., Chen C.S., Wu X., Hamm L.L., He J. (2008). Alcohol consumption and the risk of end-stage renal disease among Chinese men. Kidney Int..

[117] Joo Y.S., Koh H., Nam K.H., Lee S., Kim J., Lee C., Yun H.R., Park J.T., Kang E.W., Chang T.I. (2020). Alcohol Consumption and Progression of Chronic Kidney Disease: Results from the Korean Cohort Study for Outcome in Patients with Chronic Kidney Disease. Mayo Clin. Proc..

[118] Roerecke M., Kaczorowski J., Tobe S.W., Gmel G., Hasan O.S.M., Rehm J. (2017). The effect of a reduction in alcohol consumption on blood pressure: A systematic review and meta-analysis. Lancet Public Health.

[119] Adrogué H.J., Madias N.E. (2007). Sodium and potassium in the pathogenesis of hypertension. N. Engl. J. Med..

[120] Dasinger J.H., Fehrenbach D.J., Abais-Battad J.M. (2020). Dietary Protein: Mechanisms Influencing Hypertension and Renal Disease. Curr. Hypertens. Rep..

[121] Kalantar-Zadeh K., Kramer H.M., Fouque D. (2020). High-protein diet is bad for kidney health: Unleashing the taboo. Nephrol. Dial. Transplant..

[122] Ko G.J., Rhee C.M., Kalantar-Zadeh K., Joshi S. (2020). The Effects of High-Protein Diets on Kidney Health and Longevity. J. Am. Soc. Nephrol..

